# Prolonged aura or status epilepticus? Unmasking a first‐time migraine attack

**DOI:** 10.1002/epd2.70074

**Published:** 2025-09-08

**Authors:** Tiago Lerda Casaccia, Michael Unterhofer, Tobias Moser, Markus Leitinger, Giorgi Kuchukhidze, Eugen Trinka, Pilar Bosque Varela

**Affiliations:** ^1^ Department of Neurology, Neurocritical Care and Neurorehabilitation, Christian Doppler University Hospital, Centre for Cognitive Neuroscience, Member of the European Reference Network EpiCARE Paracelsus Medical University of Salzburg Salzburg Austria; ^2^ Neuroscience Institute, Centre for Cognitive Neuroscience Christian Doppler University Hospital Salzburg Austria; ^3^ Karl Landsteiner Institute for Neurorehabilitation and Space Neurology Salzburg Austria

**Keywords:** aphasia, EEG, lateralized rhythmic delta activity, non‐convulsive status epilepticus

Migraine is one of the most common neurological disorders and ranks as one of the most disabling chronic conditions worldwide.^1^ Despite this, over half of migraine cases remain undiagnosed,^2^ often leading to inadequate or entirely absent treatment. The aura phase, present in one third of patients,^3^ typically consists of transient focal neurological deficits that last less than 60 min and precede the headaches. However, it is now known that the aura can be more variable: It may last longer than an hour and can also occur during or even after the headache.^4^ Such nonclassical aura presentations can resemble other neurologic conditions and pose diagnostic challenges.

We report the case of a 25‐year‐old previously healthy man who presented with a first‐time migraine attack with prolonged aura, mimicking non‐convulsive status epilepticus (NCSE). The patient developed sudden speech difficulties at the workplace over the course of 1 h without impairment of consciousness and was brought to the emergency room at around noon. Clinical examination revealed sensory aphasia characterized by partially fluent, hesitant, incomprehensible speech, and impaired comprehension. The rest of the examination was unremarkable. Vital signs and laboratory tests consisting of full blood and urine workups, including a complete urine toxicological panel (namely opioids, ethanol, barbiturates, benzodiazepines, amphetamines, cannabis, cocaine, and phencyclidine) were normal.

MRI showed no structural abnormalities suggestive of acute or recent cerebral ischemia (Figure 1). Electroencephalography (EEG) revealed continuous lateralized rhythmic delta activity (LRDA; 95% prevalence) in the left fronto‐centro‐temporal region with fluctuating frequency between 2.5 and 3 Hz (Figure 2A). Aphasia persisted during the EEG, amounting to a total duration of approximately 3 h since symptom onset. The administration of lorazepam 4 mg and levetiracetam 3000 mg IV produced no clinical improvement and only a slight reduction of LRDA frequency in EEG (Figure 2B). The pattern was compatible with an ictal‐interictal continuum (IIC) according to the American Clinical Neurophysiological Society criteria (ACNS),^5^ corresponding to a possible NCSE in the Salzburg diagnostic criteria of NCSE.^6^

**FIGURE 1:**
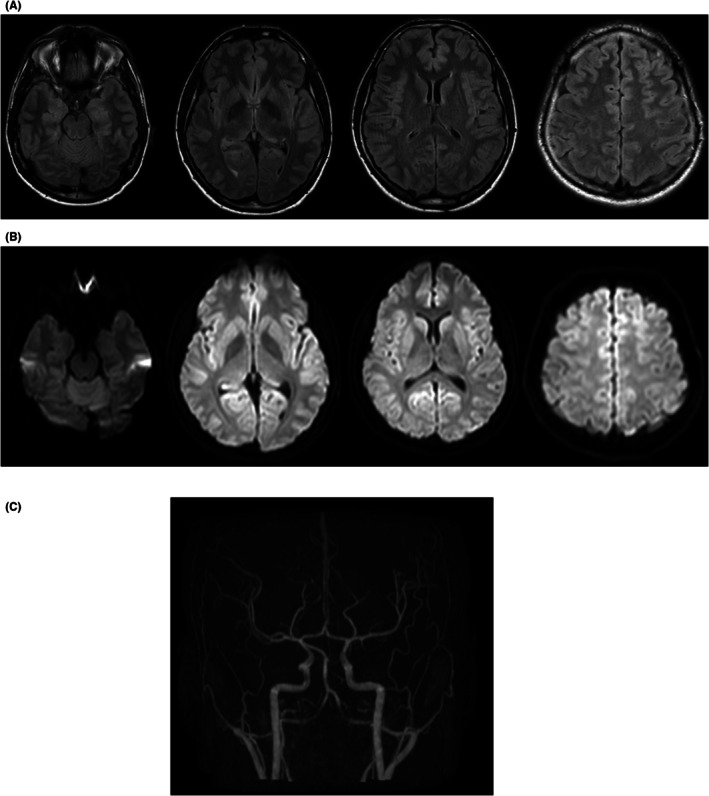
MRI without contrast was performed following the admission to the emergency room to rule out a stroke. We present progressive slices from ventral to dorsal from fluid attenuated inversion recovery (FLAIR) (A) and diffusion weighted imaging (DWI) B1000 (B) sequences, as well as Time‐of‐Flight (ToF) angiography of intracranial vessels (C), without pathological findings.

**FIGURE 2:**
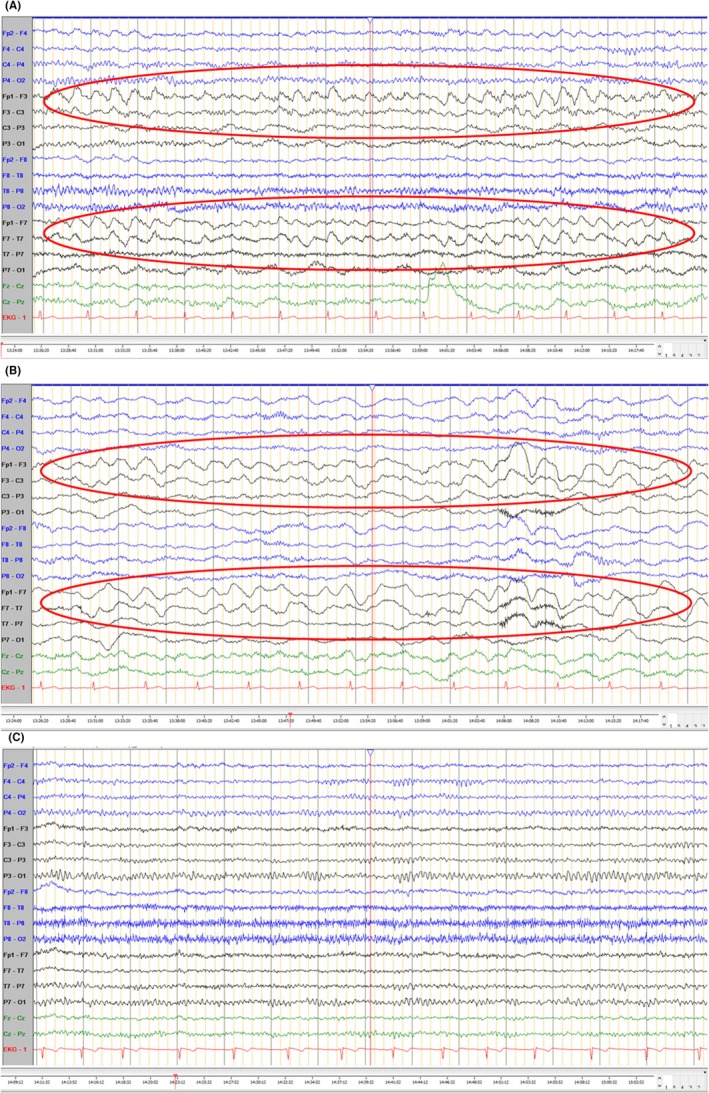
Fragments of EEG recordings during the acute phase before treatment with anti‐seizure medications showing LRDA fluctuating between 2.5 Hz and 3.0 Hz with a prevalence of 95% of a 20‐min recording (A), after administration of anti‐seizure medications (B), and during the follow‐up 3 weeks later (C). (A) The patient was aphasic and presented with continuous lateralized rhythmic delta activity (LRDA) on the fronto‐centro‐temporal region (more prominent on Fp1‐F3, F3‐C3, Fp1‐F7, and F7‐T7; highlighted with red ovals) with a fluctuating frequency between 1.5 and 3 Hz. (B) After administration of 3000 mg of levetiracetam and 4 mg of lorazepam, there was no clinical improvement and only a slight decrease in rhythmicity shown on the EEG (highlighted with red ovals), which was compatible with a possible non‐convulsive status epilepticus (NCSE). (C) During a follow‐up 3 weeks later, when the patient was asymptomatic, no relevant EEG changes were found.

After the aphasia resolved the following day, the patient described a severe headache and progressive numbness spreading from the right hand to the right arm and face, preceding the aphasia. He also reported a scotoma before the headache and a family history of migraines. Based on this, nonsteroidal anti‐inflammatory medication was administered, rendering him symptom‐free. Continuous EEG monitoring showed no further LRDA, and he was discharged with a diagnosis of first‐time migraine with prolonged aura. Three weeks later, the patient continued symptom‐free, and EEG was unremarkable (Figure 2C).

EEG abnormalities have been reported during migraine aura, similar to the ones presented in this case, in the form of bifrontal rhythmic delta activity and intermittent lateralized delta–theta activity.^7^ The most consistent findings have been observed in patients with familial hemiplegic migraine and migraine with brainstem aura, typically involving diffuse or lateralized slowing.^8^ This EEG activity may be related to the underlying cortical spreading depression (CSD), a phenomenon first described in animal models by Leão in 1944.^9^ CSD arises from complex changes in neuronal activity, vascular function, and extracellular ion concentrations triggered by neuronal damage,^10^ consisting of a sudden depolarization and a subsequent wave of suppression of cortical activity. Although it is the most accepted explanation for the pathophysiology of migraine aura,^7^, ^8^ it has not been definitively recorded with surface EEG. The high impedances of the skull and dura, paired with the limited spatial discrepancy of surface EEG, make it unlikely to detect the slow and discrete changes of CSD.^10^

Evidence has been produced using intracranial electrodes in conditions such as stroke and intracranial hemorrhage,^10^ while recently also in a patient experiencing migraine aura.^11^ A novel prospective study demonstrated a consistent reduction in alpha power on high‐density EEG during visual aura,^12^ which remains nonspecific and cannot yet be considered definitive evidence of CSD.

The patient's initial presentation was suggestive of NCSE, by the sudden onset of focal neurological symptoms and fluctuating LRDA on EEG.^13^ Although the Salzburg criteria demonstrate high sensitivity and specificity,^6^ they also allow for the classification of a broader group of patients under the category of “possible NCSE” to minimize the risk of underdiagnosing. In these cases, differential diagnoses should be considered, highlighting the critical importance of clinical assessment before applying the criteria.

## FUNDING INFORMATION

This work received no external funding.

## CONFLICT OF INTEREST STATEMENT

Eugen Trinka reports personal fees from Biogen, Novartis, Roche, Sanofi, EVER Pharma, Marinus, Arvelle, Angelini, Argenx, Medtronic, Biocodex Bial‐Portela & Cª, NewBridge, GL Pharma, GlaxoSmithKline, Boehringer Ingelheim, LivaNova, Eisai, Epilog, UCB, Biogen, Sanofi, Jazz Pharmaceuticals, and Actavis, none of them related to the presented work. His institution received grants from Biogen, UCB Pharma, Eisai, Red Bull, Merck, Bayer, the European Union, FWF Österreichischer Fond zur Wissenschaftsförderung, Bundesministerium für Wissenschaft und Forschung, and Jubiläumsfond der Österreichischen Nationalbank, none of them related to the presented work. Tobias Moser reports personal fees from Biogen, BMS, Novartis, Roche, Sanofi, Merck, and Teva, none of them related to the presented work. Giorgi Kuchukhidze received research grants from Austrian Research Fund (FWF), travel grants, and honoraria from UCB, Jazz Pharmaceuticals, and Novartis, none of them related to the presented work. Pilar Bosque Varela received travel grants and honoraria from UCB. The rest of the authors do not have disclosures.

## PATIENT CONSENT STATEMENT

Written informed consent was obtained from the patient.

**Test yourself**

1. Which of the following EEG patterns can be detected in migraine patients during the aura phase?
  - Triphasic waves
  - Lateralized rhythmic delta activity
  - Burst suppression
  - Periodic discharges
2. The diagnosis of non‐convulsive status epilepticus:
  - Is exclusively electrophysiological
  - Is only valuable in patients with known epilepsy history
  - Should not be applied in patients with migraine
  - Requires an integration of clinical and electrophysiological data
3. Which of the following statements is correct regarding cortical spreading depression?
  - It is a phenomenon exclusive to migraine
  - It has only been described in human beings
  - It is the main pathophysiological explanation for migraine aura
  - It is easily recorded by scalp EEG

*Answers may be found in the* *supporting information*.

## Supporting information


Data S1.

Data S2.

## Data Availability

The data that support the findings of this study are available on request from the corresponding author. The data are not publicly available due to privacy or ethical restrictions.
