# Supplementary material for: Prolonged aura or status epilepticus? Unmasking a first‐time migraine attack
Source: Epileptic Disord. 2025 Sep 8;27(5):1060–5. doi: 10.1002/epd2.70074 (PMC12574488; doi:10.1002/epd2.70074)
Supplement: Supplementary file 1 — Data S1. [file EPD2-27-1060-s001.pptx]

## Slide 1
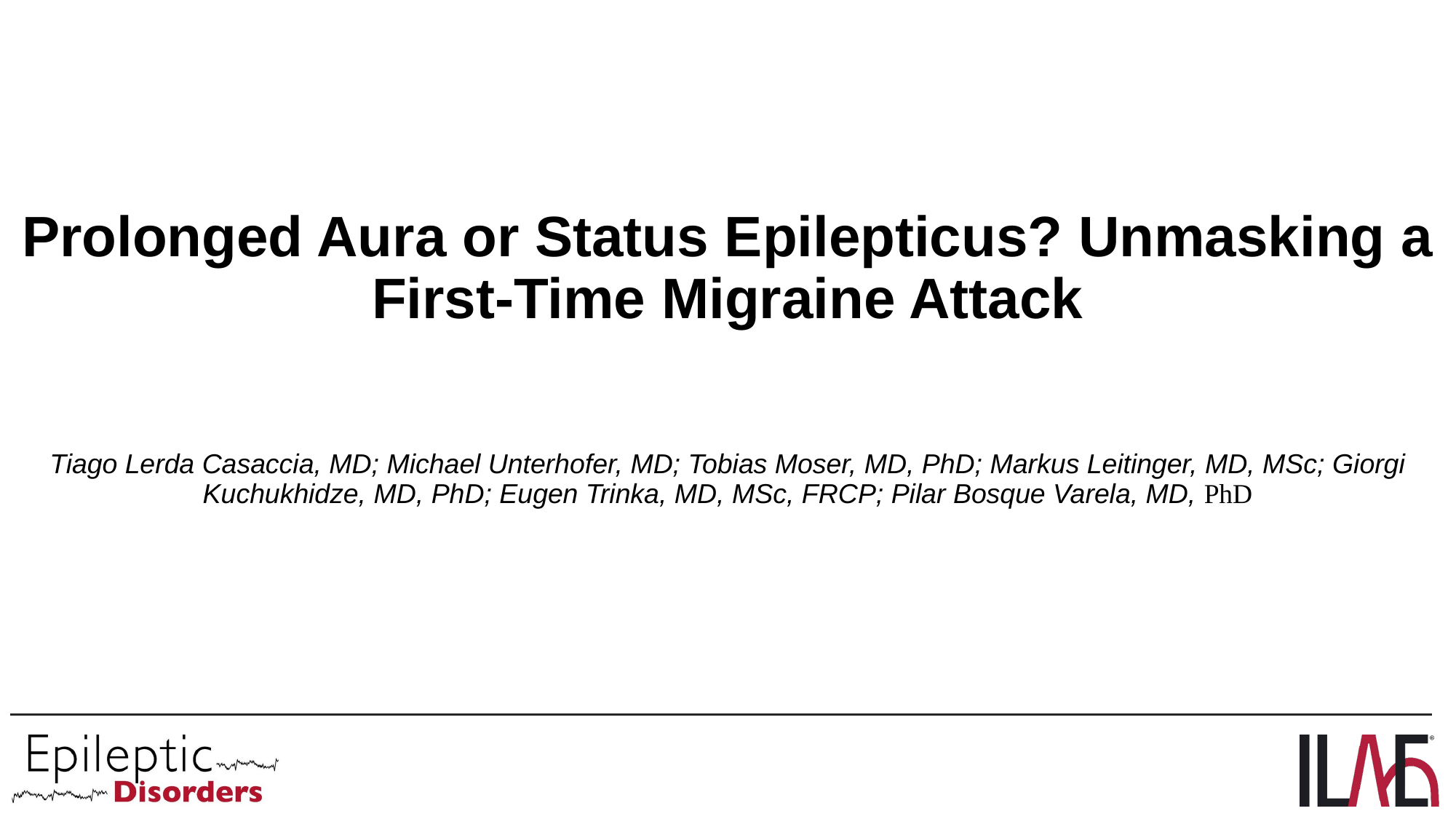

# Prolonged Aura or Status Epilepticus? Unmasking a First-Time Migraine Attack
Tiago Lerda Casaccia, MD; Michael Unterhofer, MD; Tobias Moser, MD, PhD; Markus Leitinger, MD, MSc; Giorgi Kuchukhidze, MD, PhD; Eugen Trinka, MD, MSc, FRCP; Pilar Bosque Varela, MD, PhD

## Slide 2
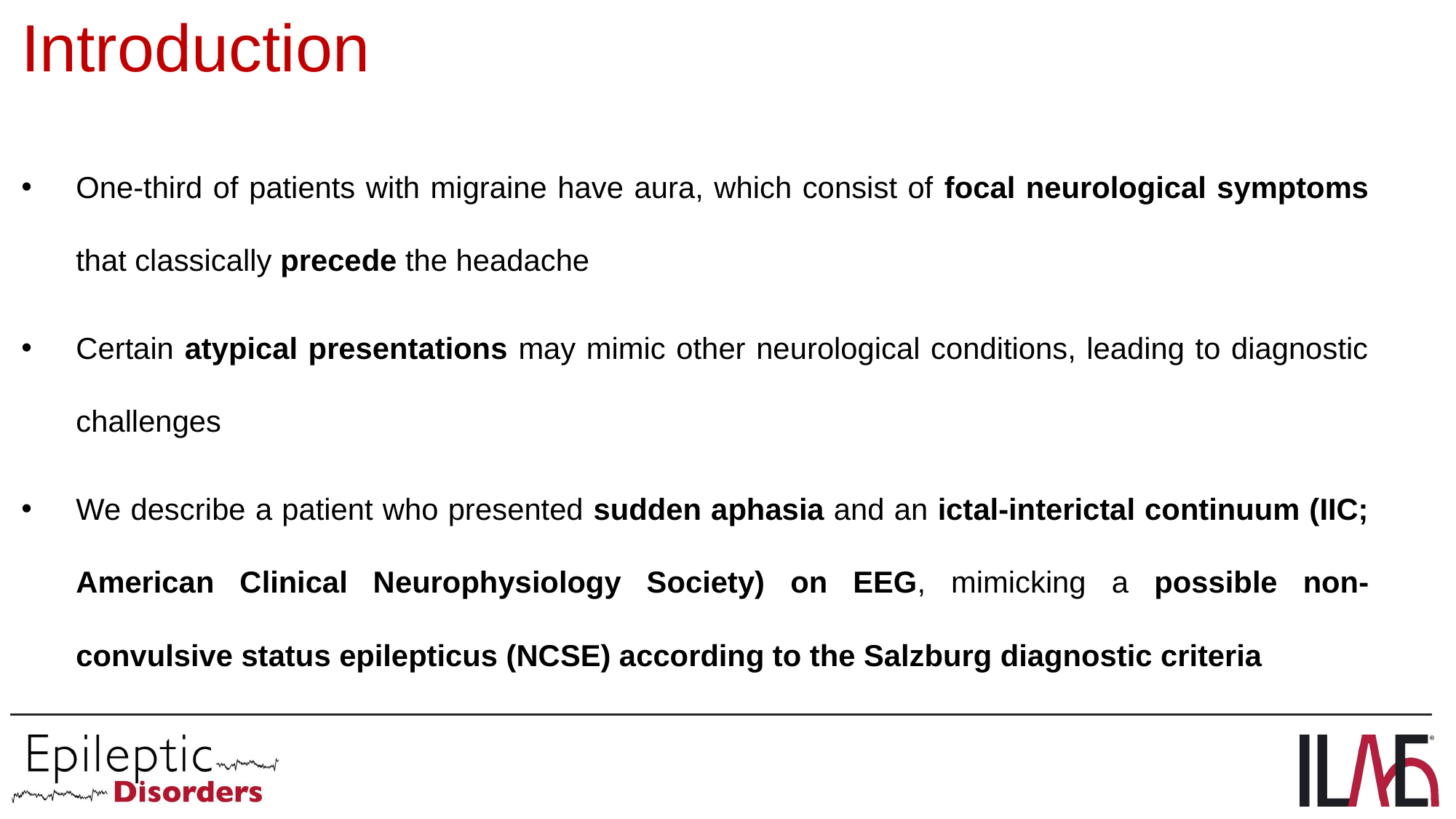

Introduction
One-third of patients with migraine have aura, which consist of focal neurological symptoms that classically precede the headache
Certain atypical presentations may mimic other neurological conditions, leading to diagnostic challenges
We describe a patient who presented sudden aphasia and an ictal-interictal continuum (IIC; American Clinical Neurophysiology Society) on EEG, mimicking a possible non-convulsive status epilepticus (NCSE) according to the Salzburg diagnostic criteria

## Slide 3
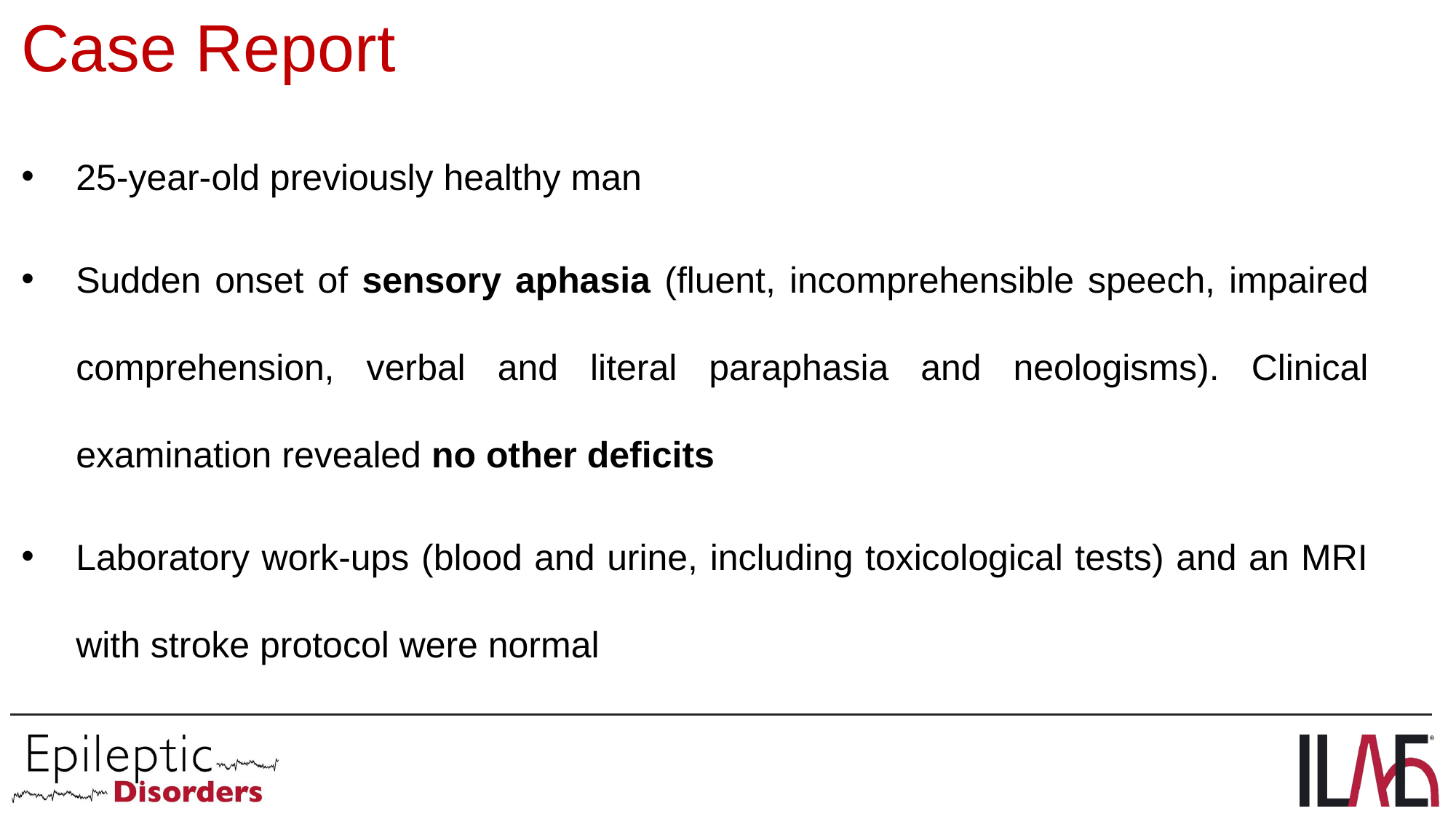

Case Report
25-year-old previously healthy man
Sudden onset of sensory aphasia (fluent, incomprehensible speech, impaired comprehension, verbal and literal paraphasia and neologisms). Clinical examination revealed no other deficits
Laboratory work-ups (blood and urine, including toxicological tests) and an MRI with stroke protocol were normal

## Slide 4
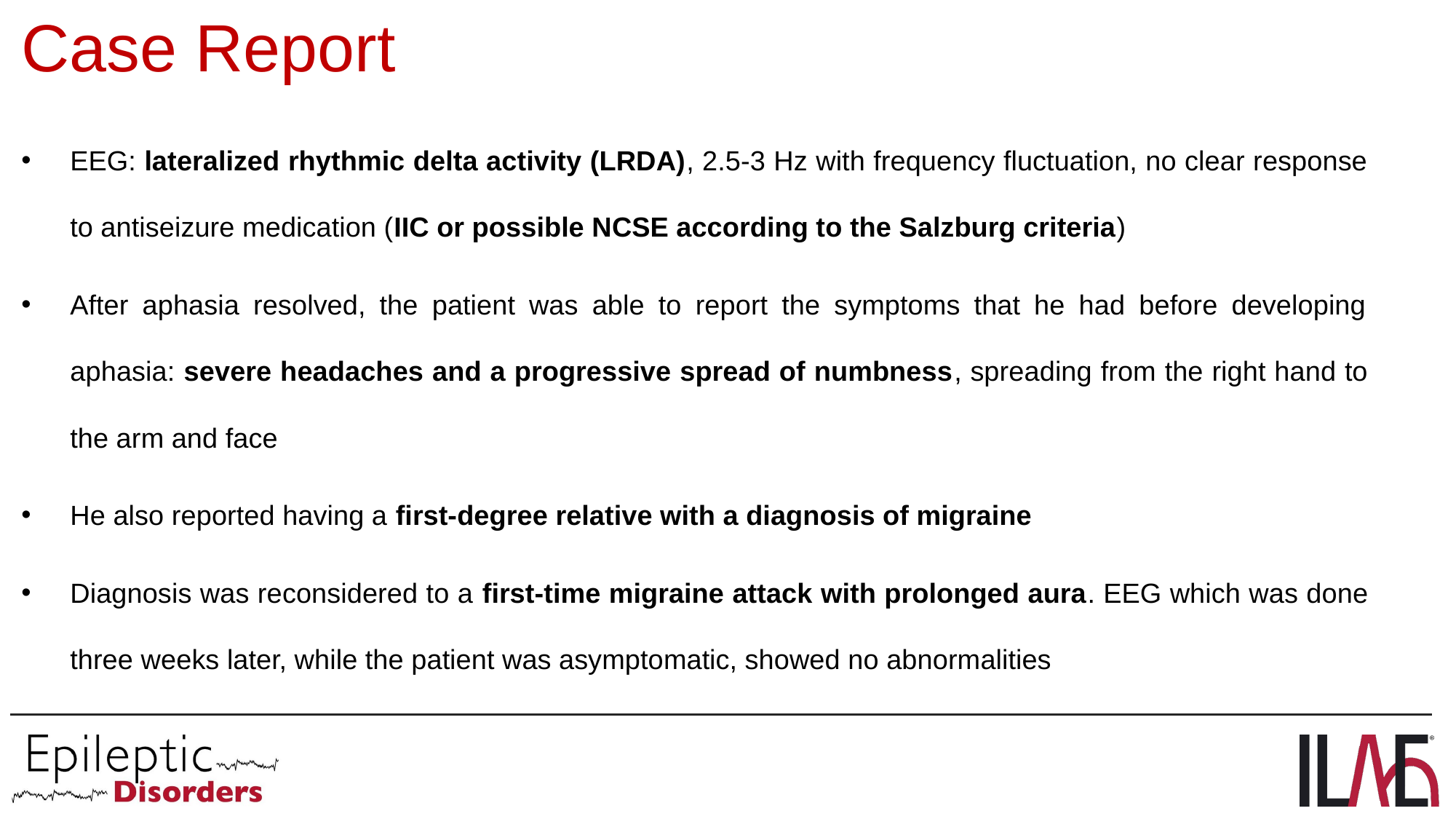

Case Report
EEG: lateralized rhythmic delta activity (LRDA), 2.5-3 Hz with frequency fluctuation, no clear response to antiseizure medication (IIC or possible NCSE according to the Salzburg criteria)
After aphasia resolved, the patient was able to report the symptoms that he had before developing aphasia: severe headaches and a progressive spread of numbness, spreading from the right hand to the arm and face
He also reported having a first-degree relative with a diagnosis of migraine
Diagnosis was reconsidered to a first-time migraine attack with prolonged aura. EEG which was done three weeks later, while the patient was asymptomatic, showed no abnormalities

## Slide 5
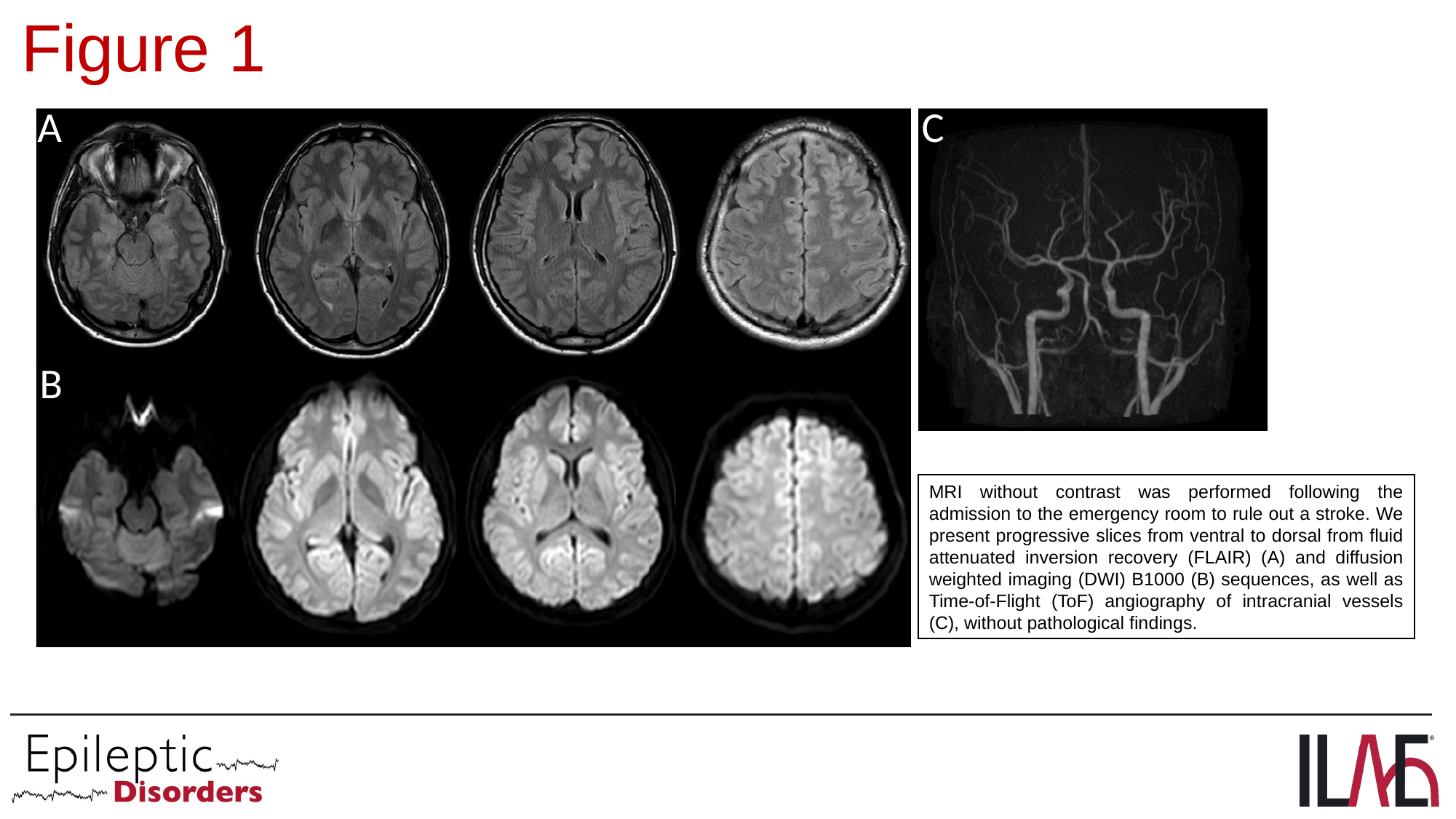

Figure 1
A
C
B
MRI without contrast was performed following the admission to the emergency room to rule out a stroke. We present progressive slices from ventral to dorsal from fluid attenuated inversion recovery (FLAIR) (A) and diffusion weighted imaging (DWI) B1000 (B) sequences, as well as Time-of-Flight (ToF) angiography of intracranial vessels (C), without pathological findings.

## Slide 6
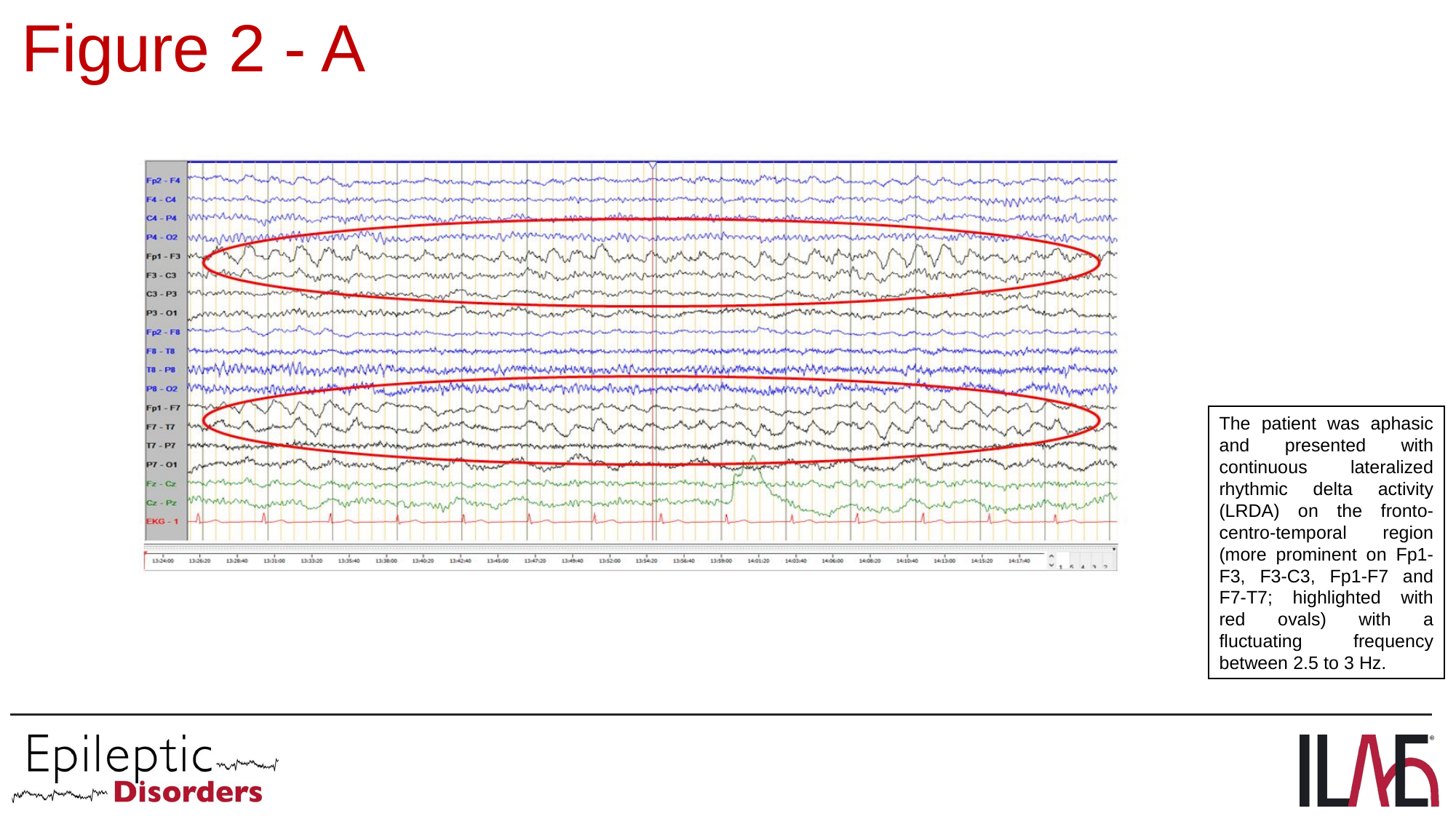

Figure 2 - A
A
C
B
The patient was aphasic and presented with continuous lateralized rhythmic delta activity (LRDA) on the fronto-centro-temporal region (more prominent on Fp1-F3, F3-C3, Fp1-F7 and F7-T7; highlighted with red ovals) with a fluctuating frequency between 2.5 to 3 Hz.

## Slide 7
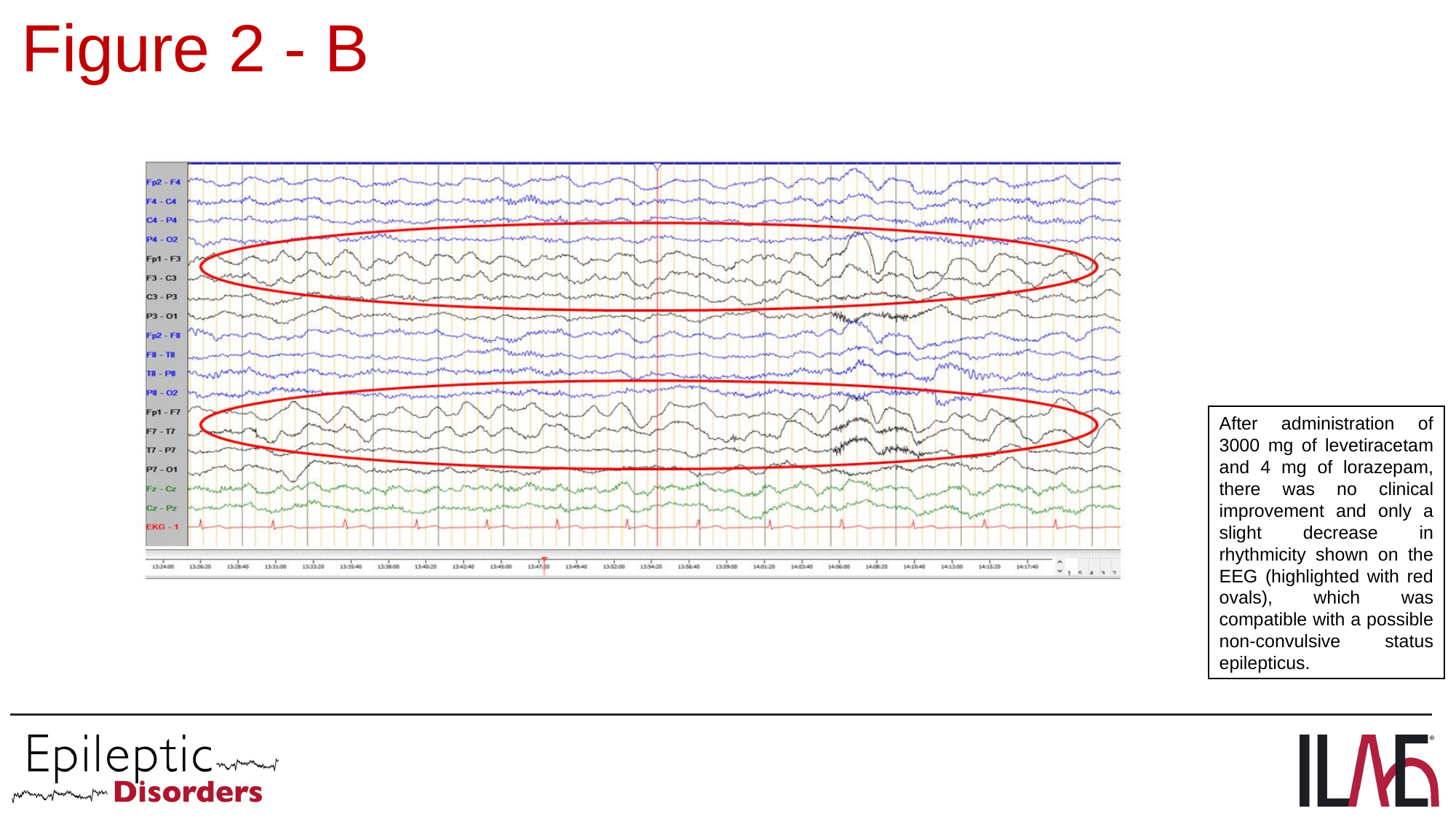

Figure 2 - B
A
C
B
After administration of 3000 mg of levetiracetam and 4 mg of lorazepam, there was no clinical improvement and only a slight decrease in rhythmicity shown on the EEG (highlighted with red ovals), which was compatible with a possible non-convulsive status epilepticus.

## Slide 8
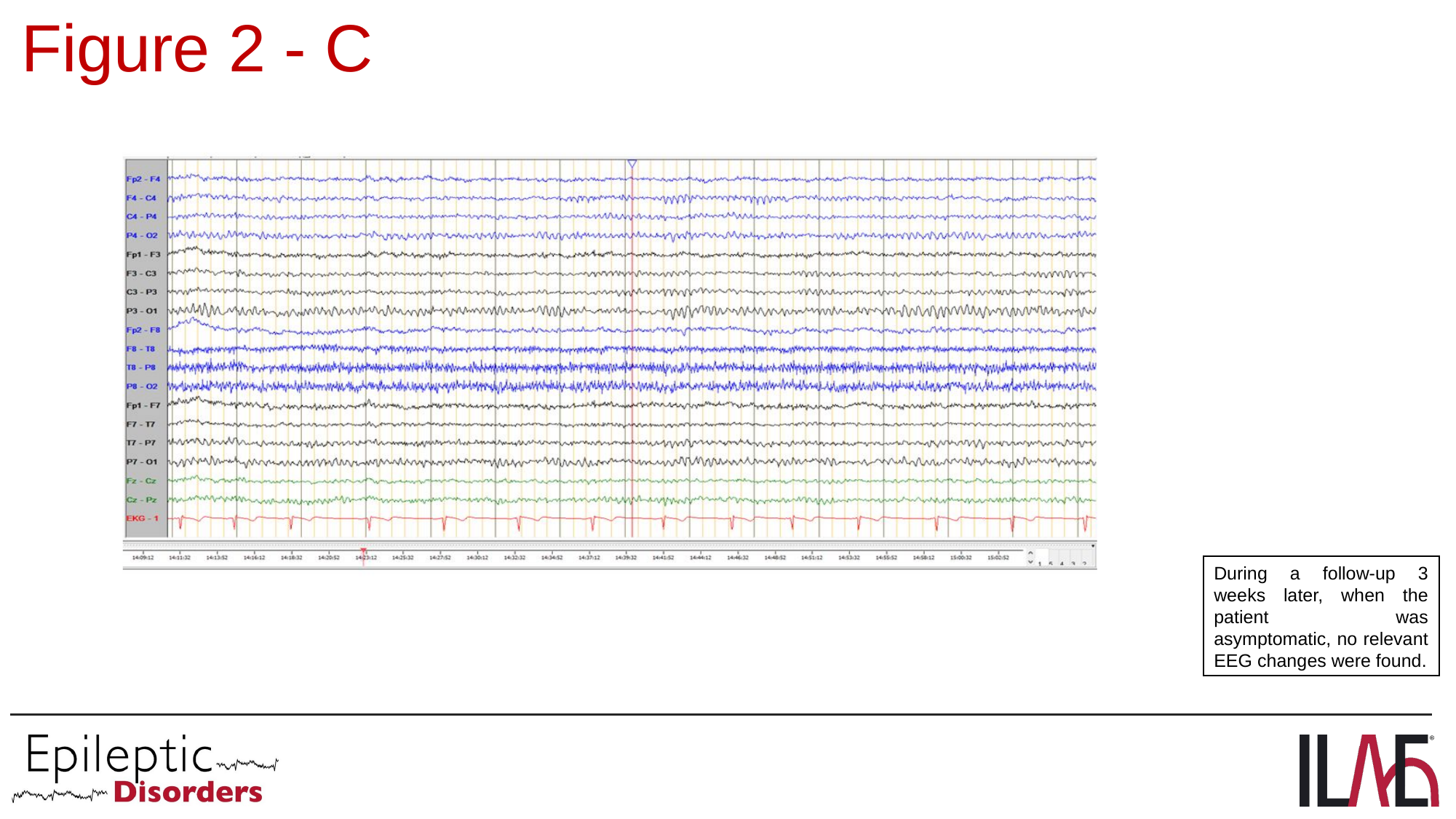

Figure 2 - C
A
C
B
During a follow-up 3 weeks later, when the patient was asymptomatic, no relevant EEG changes were found.

## Slide 9
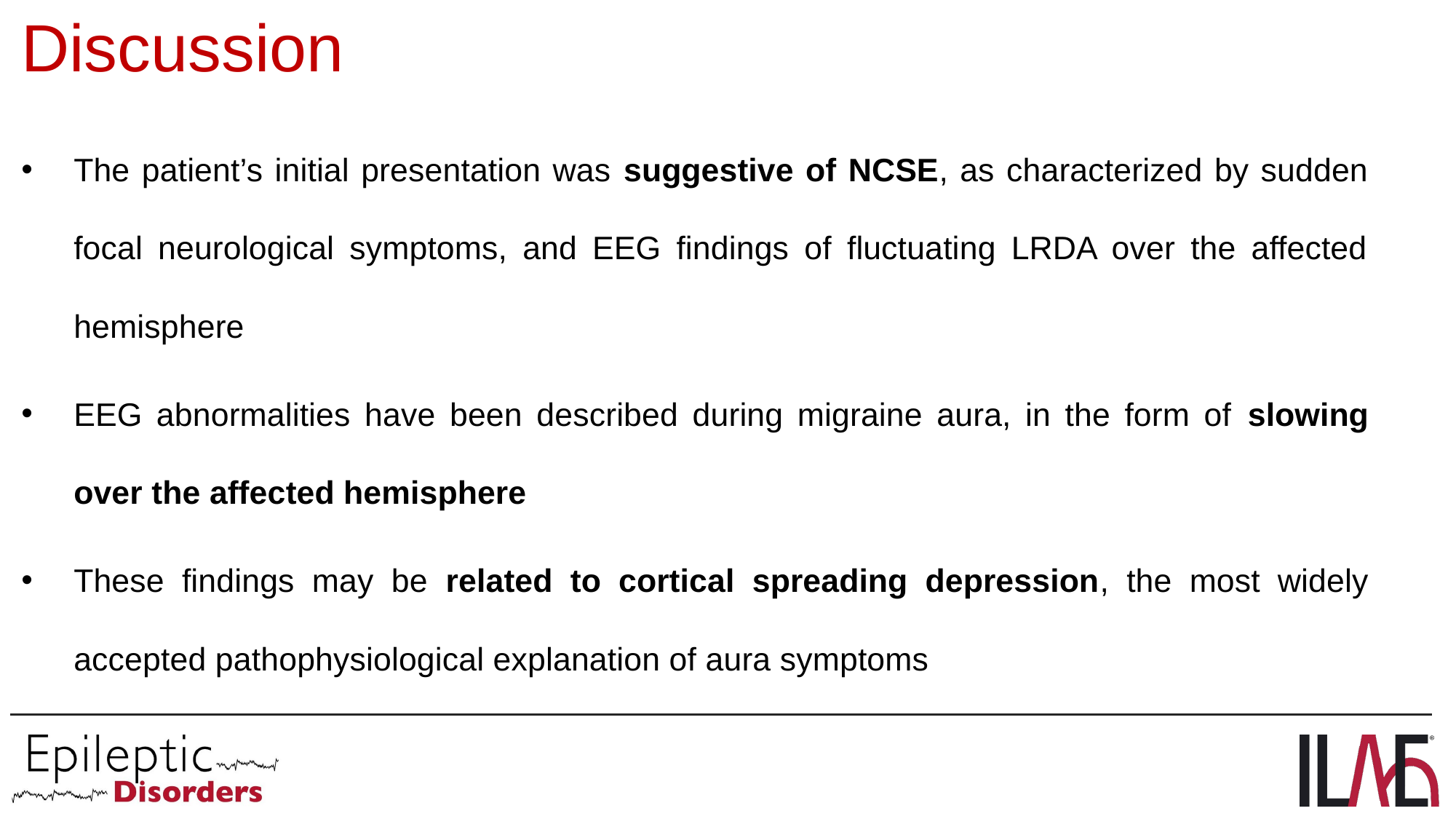

Discussion
The patient’s initial presentation was suggestive of NCSE, as characterized by sudden focal neurological symptoms, and EEG findings of fluctuating LRDA over the affected hemisphere
EEG abnormalities have been described during migraine aura, in the form of slowing over the affected hemisphere
These findings may be related to cortical spreading depression, the most widely accepted pathophysiological explanation of aura symptoms

## Slide 10
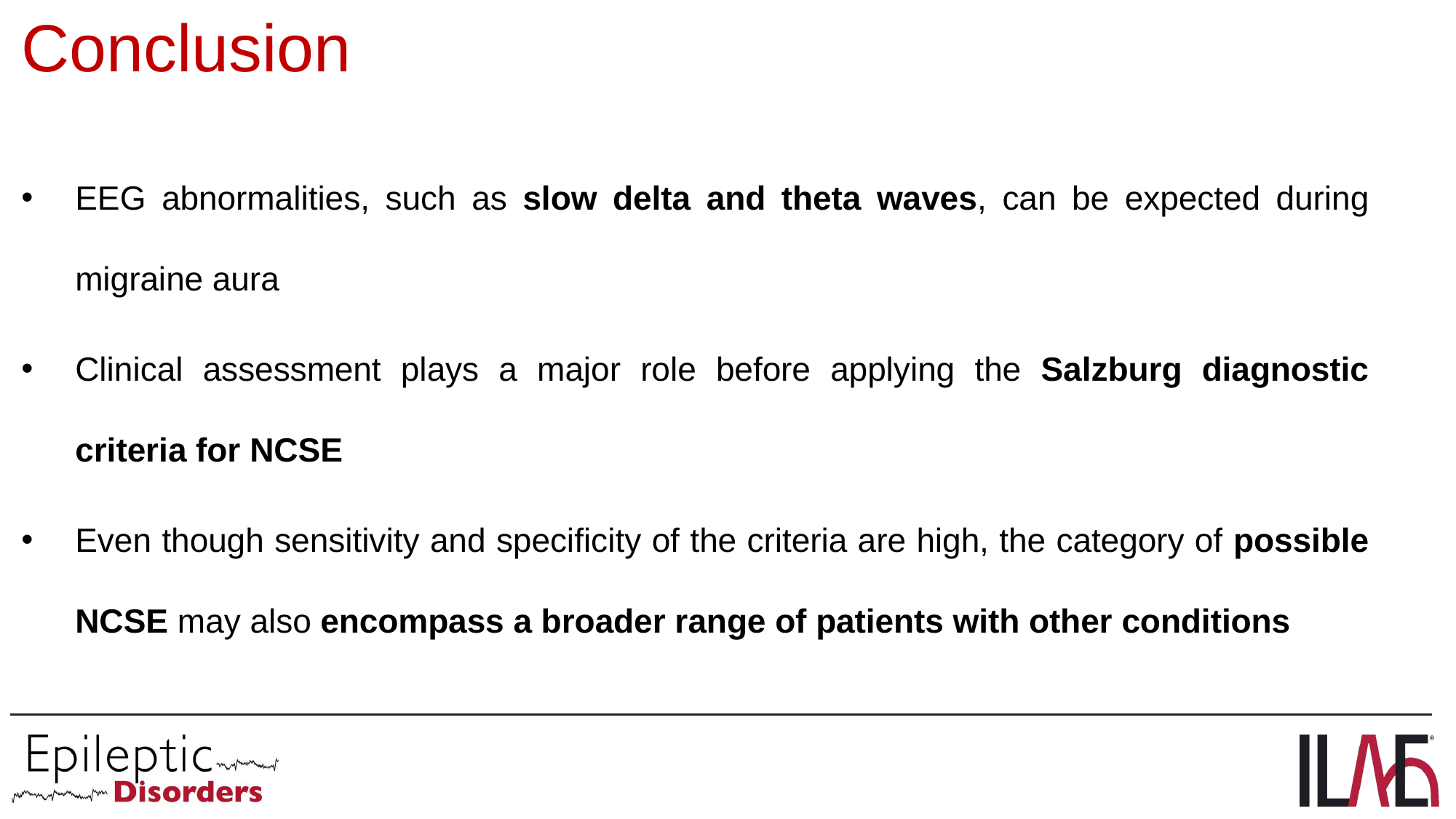

Conclusion
EEG abnormalities, such as slow delta and theta waves, can be expected during migraine aura
Clinical assessment plays a major role before applying the Salzburg diagnostic criteria for NCSE
Even though sensitivity and specificity of the criteria are high, the category of possible NCSE may also encompass a broader range of patients with other conditions
