# Supplementary material for: Prolonged aura or status epilepticus? Unmasking a first‐time migraine attack
Source: Epileptic Disord. 2025 Sep 8;27(5):1060–5. doi: 10.1002/epd2.70074 (PMC12574488; doi:10.1002/epd2.70074)
Supplement: Supplementary file 2 — Data S2. [file EPD2-27-1060-s002.docx]

Answers

1. B. Lateralized rhythmic delta activity.

2. D. Requires an integration of clinical and electrophysiological data.

3. C. It is the main pathophysiological explanation for migraine aura.
